# People, places, and things: The impact of scene animacy on emotional modulation of the early posterior negativity

**DOI:** 10.3758/s13415-026-01446-w

**Published:** 2026-05-06

**Authors:** Han Jia, Andrew H. Farkas, Dean Sabatinelli

**Affiliations:** 1 Department of Psychology, University of Georgia, Athens, GA USA; 2 Department of Psychology, University of Florida, Gainesville, FL USA

**Keywords:** Emotion, Event-related potential, Animacy, Scene perception, Late positive potential, Early posterior negativity

## Abstract

The early posterior negativity (EPN) is a mid-latency event-related potential (ERP) component reliably enhanced by emotionally arousing visual cues. Recent work suggests that modulation of the EPN might depend to some extent on evocative cues featuring animate content. We tested this possibility by recording EEG while 80 participants viewed pleasant, neutral, and unpleasant scenes depicting people, objects, or landscapes. People and object scenes were selected to be comparable in composition and arousal ratings, to enable a direct assessment of the impact of scene animacy, separate from emotional intensity. Results showed robust EPN modulation by emotional content across both people and object scenes, with no significant interaction across arousal-matched scenes. This finding further dissociates the EPN from proximal event-related potential components associated with face and body perception and supports its value as an early marker of emotional perception, reliably driven by emotional intensity across multiple domains of visual cues.

A well-studied event-related potential associated with human emotional processing is the early posterior negativity (EPN). Concurrent with the positive-going P2 component, the EPN appears as a negative voltage shift over left and right lateral occipital sensors roughly 150 to 300 ms after scene onset (Junghöfer et al., 2001; Schupp et al., 2004). The amplitude of the EPN has been shown to be enhanced by emotionally arousing (pleasant and unpleasant), relative to neutral content (Olofsson et al., 2008; Pastor et al., 2008; Schupp et al., 2006). This arousal effect can be reliably found during naturalistic scene perception and is also evident during the perception of emotional faces (Herbert et al., 2013; Jaworska et al., 2012; Langeslag et al., 2018), hand gestures (Flaisch et al., 2009), and to a lesser degree, words (Kissler & Herbert, 2013; Kissler et al., 2007; Schindler & Kissler, 2016).

Several EPN studies have reported enhanced EPN modulation in response to erotic, relative to mutilation scenes (De Cesarei & Codispoti, 2006; Flaisch et al., 2008; Frank & Sabatinelli, 2019; Schupp et al., 2004; Weinberg & Hajcak, 2010), although the experimental designs did not always employ equivalently arousing pleasant and unpleasant scenes, or control for scene complexity. To assess the potential role of exposed bodies (Downing et al., 2001) on this apparent erotica bias, two studies investigated the impact of scenes featuring upright, unclothed, but nonsexual people (nudists) and found that the EPN was more strongly modulated by nudist scenes than by erotica, despite the fact that nudist scenes receive modest arousal ratings (Farkas et al., 2020; Farkas & Sabatinelli, 2023).. This unexpected finding is inconsistent with the general interpretation that the EPN is primarily associated with rated arousal (Olofsson et al., 2008; Schupp et al., 2004).

This effect also distinguishes the EPN from the emotion-modulated late positive potential (LPP), a centroparietal slow-wave component beginning ~ 400 ms after scene onset, which correlates strongly with scene arousal ratings (Cuthbert et al., 2000; Frank & Sabatinelli, 2019; Weinberg & Hajcak, 2010). The sensitivity of the EPN to unclothed, upright bodies suggests that modulation of the EPN may depend to some extent on the presence of animate subjects in scenes, independent of emotion, perhaps as part of an early stage of visual perception that supports animacy discrimination (Altman et al., 2016; Caramazza & Shelton, 1998; McCarthy & Warrington, 1988; Thorpe et al., 1996). The EPN also overlaps somewhat with the face-evoked N170 in scalp topography and latency (Bentin et al., 1996; Carretie et al., 2013; Eimer, 2011; Rossion & Jacques, 2012), and a similar body-sensitive ERP component (Alho et al., 2015; Hietanen et al., 2014), which could also contribute to a sensitivity of the EPN to the presence of people in naturalistic scenes. There are several ways that the role of animacy in emotional perception can be investigated, but we chose first to address whether the EPN can be modulated by emotional scenes that exclude animate subjects completely, although this presents an experimental design challenge.

In human studies of emotional scene perception, the positive relationship between scene animacy and ratings of emotional arousal is well established, as depictions of predatory threat or sexual opportunity consistently evoke the greatest emotional experience in addition to peripheral and central nervous system reactivity (Bradley et al., 2001; Frank & Sabatinelli, 2019; Weinberg & Hajcak, 2010). The composition of the International Affective Picture System (IAPS; Lang et al., 2008), commonly used to evoke emotion in laboratory settings, reflects the significance of animacy in evoking emotion, with 72% of the IAPS depicting people and animals, and 28% depicting objects and landscapes (Lang et al., 2008). When the IAPS is divided by animate and inanimate content and further subdivided by valence (pleasant, neutral, and unpleasant), normative valence ratings are roughly equivalent across animate and inanimate scenes, while arousal ratings are comparatively enhanced for animate scenes regardless of emotional content (Lang et al., 2008). In other words, scenes featuring living things are rated as more arousing irrespective of whether they are neutral or emotional. While the impact of scene animacy on the basic processes of visual system discrimination has received considerable investigation (Harel et al., 2011; McClelland et al., 2014; Sha et al., 2015; Vlcek et al., 2020; Watson & Andrews, 2024), these studies rarely depict content that could be described as emotionally evocative, and thus the effects of animacy on emotional scene perception has not been well described.

While emotional modulation of the EPN has been repeatedly shown to be associated with rated scene arousal, whether pleasant or unpleasant, it could be that the effects identified in a given study are driven in part by a greater proportion of animate subjects in the pleasant and/or unpleasant relative to neutral scene stimuli. In fact, it is common for experimental scene sets to be assembled that include a higher proportion of objects depicted in neutral categories than in emotional categories (Brazdil et al., 2009; Cuthbert et al., 2000; Foti et al., 2009; Solomon et al., 2012).

To address this research question, pleasant and unpleasant object scenes were collected that depicted the most evocative content available. To minimize a potential confound with arousal ratings, pleasant and unpleasant people scenes were collected that, based on past studies, would be expected to evoke moderate ratings of arousal, thus facilitating a comparison of ERP modulation between equivalently arousing animate and inanimate scenes. Neutral objects and people scenes were selected to enable a comparison of potential animacy-related ERP modulation, independent of emotion. To enhance homogeneity in our stimulus set, we did not include scenes depicting animals, and thus our comparison of animate versus inanimate scenes is more specifically scenes featuring people compared with objects and landscapes. Because scene composition (simple vs. complex) has been shown to moderate the EPN independent of emotion (Bradley et al., 2007; Löw et al., 2013; Wiens et al., 2011), the stimulus set was curated to avoid systematic differences in figure/ground relationships across people and object scenes. Pleasant, neutral, and unpleasant landscapes were also included, both to expand stimulus variability and because these scene subjects are often overrepresented as neutral content in ERP studies of emotional processing, but we did not expect that arousal ratings evoked by landscape scenes could be matched with arousal ratings evoked by people or object scenes (Lang et al., 2008). The LPP was recorded to provide a reliable electrophysiological index of emotional arousal, by which modulation of the EPN could be interpreted. For example, if viewing arousing objects modulates the LPP to the same extent as viewing arousing people, we might interpret any difference we might find in EPN modulation to be specific to early stages of perceptual categorization.

If, as described above, modulation of the EPN reflects sensitivity to scene arousal, we should find enhanced EPN negativity as participants view pleasant and unpleasant scenes, regardless of content, with equivalent modulation for equivalently arousing scenes, as rated by our sample. If the EPN reflects both rated scene arousal and the presence of people, stronger modulation would be evoked by scenes depicting emotional people relative to emotional objects, despite equivalent ratings of arousal.

## Method

### Participants

This experiment employs moderately arousing scene stimuli and seeks to determine whether emotional scenes depicting people and emotional scenes depicting objects differentially modulate the EPN, a potentially subtle effect that requires sufficient statistical power to detect. Therefore, we conducted a sensitivity power analysis using G*Power 3.1.9.7, which led us to the large sample size of 80 participants, who were recruited from the UGA Psychology Department Research Pool and compensated with Psychology course credit. Of these 80 participants, 77 (53 females, mean age = 19.2, standard deviation [SD] = 2.1) were included in the final analysis. Three participants were excluded due to an excess of artifact-contaminated trials. Specifically, if the EEG processing pipeline identified an excess of artifacts (described below) in more than 50% of trials for any scene category (pleasant, neutral, or unpleasant) in any scene condition (people, landscapes, objects), that participant was excluded. Among the remaining 77 participants, self-identified race/ethnicity included 53 Whites, 12 Asians, 7 Hispanics, 1 Black, and with 4 participants choosing not to disclose their race/ethnicity. All participants gave informed consent before the study, which had been approved by the University of Georgia Human Subject Institutional Review Board. With a final sample of 77 and a 3 (Emotion) × 3 (Category) repeated-measures design, the analysis indicates 80% power (α = .05, two-tailed) to detect effects as small as Cohen’s f = 0.105 (partial η^2^ ≈ .01).

### Stimuli

**Fig. 1:**
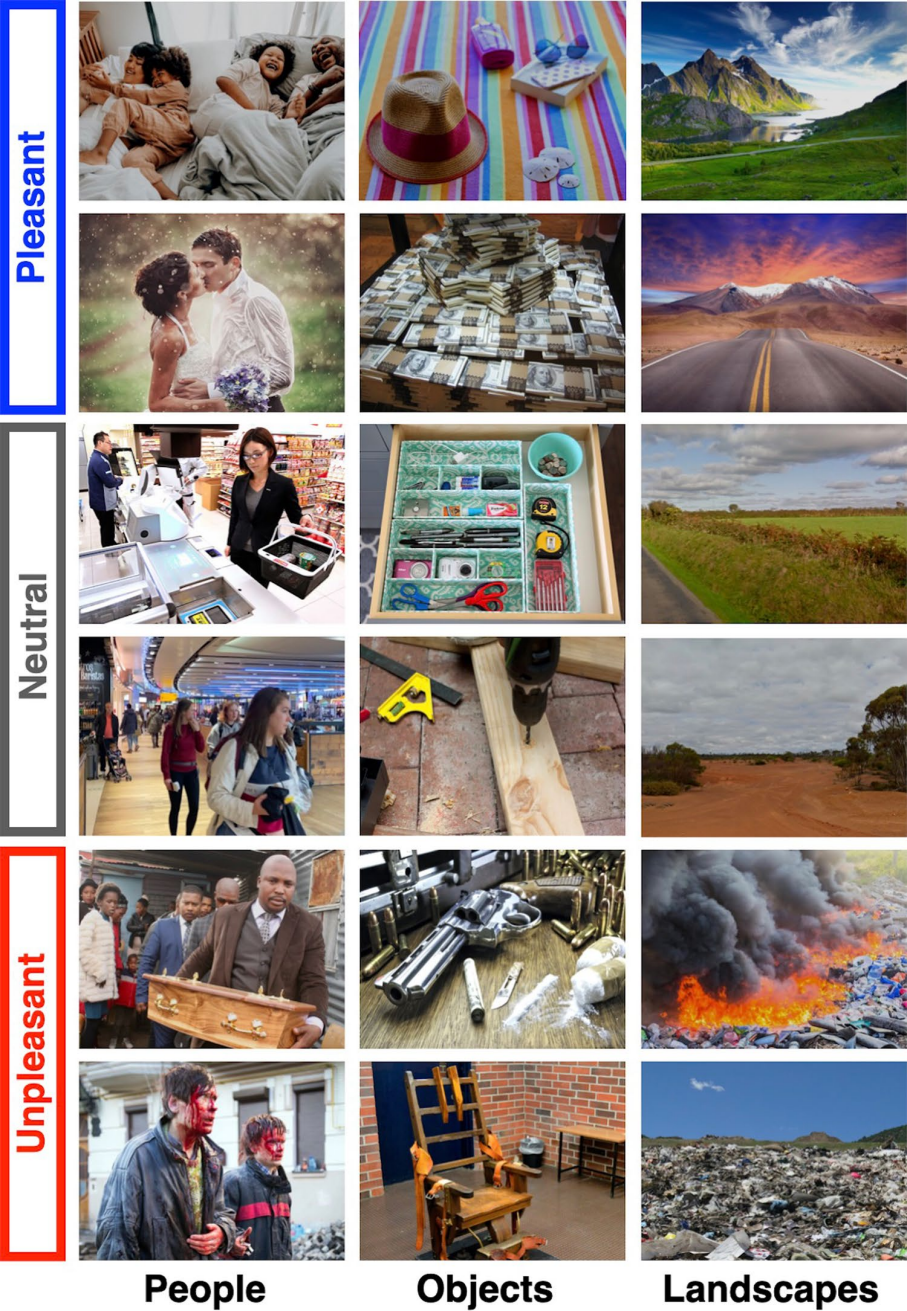
Example stimuli used in the experiment. Images were grouped by category (People, Objects, Landscapes) and valence (Pleasant, Neutral, Unpleasant)

The study utilized photographic stimuli across three emotional categories (pleasant, neutral, unpleasant) and three different contents (people, landscapes, objects) for a total of 180 scenes. Example scenes are shown in Fig. 1. The scene set was curated in our lab using uncopyrighted online sources, and balanced across the nine categories to be statistically equivalent (*p* > .2) in luminance and 90% quality JPEG file size, as a rough index of complexity (Donderi, 2006). People scenes depicted two or more individuals in various contexts, excluding explicit erotica and mutilations, to increase the likelihood of comparable arousal ratings across the scene categories. Landscape scenes depicted natural scenery without humans or animals, with the restriction that objects could not be in the immediate foreground. Object scenes depicted foreground items such as weapons, crashed cars, tools, kitchen utensils, precious metals, and currency. The great majority of object scenes depicted multiple items in an array, such as a desk drawer or tool bench. All scenes were displayed at a resolution of 1024 × 768 pixels in the middle of a 2560 × 1440 resolution LG 27GN800-B monitor set to a refresh rate of 144 Hz. The maximum vertical and horizontal angles of the scene were 11.48° and 15.31°, respectively. The monitor background was consistently gray throughout the experiment. The 180 scenes were presented twice in two successive pseudorandom sequences, resulting in 360 total trials. No more than two consecutive scenes featured the same content or emotional category within each block.

### Procedure

Participants arrived at the laboratory and, after providing consent, were seated in an electrically shielded chamber, positioned 120 cm from the monitor. The experimenter provided a verbal overview of the EEG equipment setup and the overall experimental protocol. Application of the EEG cap required 15 to 30 min. Following this, participants viewed the scene series alone in the soundproof and darkened chamber. The series began with a red fixation cross displayed against a gray background for 1.5 to 2.5 s, reminding participants to focus on the screen center. Each participant completed two blocks of 180 trials, with a short self-paced break between blocks to reduce fatigue. Scenes then appeared for 1 s each, followed by a gray screen and fixation cross for 1.5 to 2.5 s until the next scene appeared. EEG data were recorded continuously during the 30-min session.

After the experimental session, the researcher returned to remove the EEG cap, and participants were then seated in a quiet room to provide emotional ratings of all scenes using a computerized version of the Self-Assessment Manikin (SAM; Bradley & Lang, 1994). Participants used a draggable slider positioned below each scene and the corresponding SAM icon to assign their ratings, from 1 to 9. The slider was initially set at 5, and participants were required to move the slider before progressing to the next scene. The sequence of the images was randomized. The experimenter gave verbal instructions on rating the scenes on valence and arousal as they felt during their first viewing and demonstrated an example trial to ensure the process was understood. Following completion of ratings, participants filled out a brief post-experiment questionnaire to provide basic demographics and feedback to the research team. Finally, participants were debriefed, shown their EEG traces, and all questions were answered.

### EEG acquisition and processing

EEG data were recorded with a 128-channel BioSemi ActiveTwo EEG cap (BioSemi Amsterdam, Netherlands), placing the Cz sensor halfway between the nasion and inion with sensors positioned according to the 10–20 layout. The system used two common mode electrodes (Common Mode Sense and Driven Right Leg) and the data were average referenced during preprocessing. Data were recorded at a 512 Hz sample rate with a 0.16 to 100 Hz bandpass filter. Electrolyte gel was applied to each sensor cup and hair was gently moved aside with a blunted syringe until impedance values stabilized below 50 μV offset.

For the ERP analysis, EEG data were preprocessed and segmented with the EMEGS analysis package in MATLAB (emegs.org; Peyk et al., 2011). Data were filtered using a low-pass Butterworth filter with a stopband of 40 Hz and a passband of 30 Hz to control for high-frequency noise. Additionally, a high-pass Butterworth filter with a stopband of 0.05 Hz and a passband of 0.1 Hz was applied. Artifacts were identified through an automated analysis in EMEGS that utilized median values of maximum amplitude, standard deviation, and maximum first derivative to detect unusable trials and unreliable electrodes. After preprocessing was completed, the data were segmented from 100 ms before scene onset until scene offset at 1,000 ms. ERPs for each scene category and each participant were then estimated from baseline-corrected EEG time data using the 100 ms window preceding scene onset. The ERP time windows and electrode locations were chosen based on topographical visualization of the data average across all trials and to be consistent with previous studies employing the same scene perception paradigm (Farkas & Sabatinelli, 2023; Frank & Sabatinelli, 2019). The EPN amplitudes were averaged from 150–300 ms after picture onset using sensors PPO9h, PO7, PO9, POO9H, PPO10h, PO8, PO10, and POO10h. The LPP amplitudes were averaged from 400–900 ms after picture onset, using sensors CCP1h, CP1, CPz, CPP1h, Pz, CPP2h, CP2, and CPP2h. For each category, the mean number of trials contributing to the waveforms ranged from 32.4 to 34.3 (M = 33.9, SD = 3.4), out of a total possible of 40.

### Statistical analyses

All statistical analyses were conducted by using RStudio (r-project.org). Repeated-measures ANOVAs for the estimated EPN and LPP amplitudes and generalized eta squared (η^2^) values were used to calculate effect sizes, quantifying variance and comparing them across ANOVAs to assess the strength of emotional modulation. We tested sphericity with Mauchly’s test and applied Greenhouse–Geisser corrections when violated. Additionally, paired *t*-tests were conducted to compare pleasant, unpleasant, and neutral contents within each category. The same approach was used to assess differences in SAM ratings of valence and arousal across scene categories.

## Results

### Scene ratings

As expected, valence ratings differed significantly across scenes selected to depict pleasant, neutral, and unpleasant material *F*(1.36, 103.55) = 453.78, *p* < .001, *η*^*2*^ = .857. The effect of content (people, objects, landscapes) was also significant, *F*(1.79, 136.036) = 32.450, *p* < .001, *η*^*2*^ = .299. The interaction of valence and content was also significant *F*(3.55, 269.67) = 23.862,* p* < .001,* η*^*2*^ = .239. Ratings of arousal also differed significantly across all scene categories, *F*(1.77, 134.61) = 65.77, *p* < .001, *η*^*2*^ = .464. The effect of content was also significant, *F*(1.37, 104.25) = 162.59, *p* < .001, *η*^*2*^ = .681. Again, the interaction of arousal and content was significant *F*(2.48, 188.22) = 11.39,* p* < .001, *η*^*2*^ = .13. Table 1 lists the 18 individual contrasts of SAM ratings for each scene valence and content. In summary, the overall pattern of ratings was quite consistent across content, with emotional scenes rated as more arousing than neutral scenes, and pleasant scenes rated as more pleasant than neutral scenes. However, valence ratings were highest for pleasant landscapes and lowest for unpleasant people, while people scenes tended to evoke more rated arousal than object scenes, and landscape scenes were rated as the least arousing overall by a wide margin.

**Table 1:** Comparisons of SAM ratings across category within emotion

Valence					
Emotion	Content (I)	Content (J)	Difference (I-J)	Standard error	*p*
Neutral	Landscapes	Objects	.108	.116	ns
	Landscapes	People	− .055	.117	ns
	Objects	People	− .163	.058	.019
Pleasant	Landscapes	Objects	1.105	.112	< .001
	Landscapes	People	.636	.119	< .001
	Objects	People	− .469	.122	< .001
Unpleasant	Landscapes	Objects	.077	.067	ns
	Landscapes	People	.488	.074	< .001
	Objects	People	.410	.064	< .001
Arousal					
Emotion	Content (I)	Content (J)	Difference (I-J)	Standard Error	*p*
Neutral	Landscapes	Objects	− 1.258	.106	< .001
	Landscapes	People	− 1.543	.117	< .001
	Objects	People	− .284	.082	< .01
Pleasant	Landscapes	Objects	− 1.630	.227	< .001
	Landscapes	People	− 2.275	.237	< .001
	Objects	People	− .645	.125	< .001
Unpleasant	Landscapes	Objects	− .929	.091	< .001
	Landscapes	People	− 1.088	.100	< .001
	Objects	People	− .160	.066	ns

### Onset ERP modulation—early posterior negativity

**Fig. 2:**
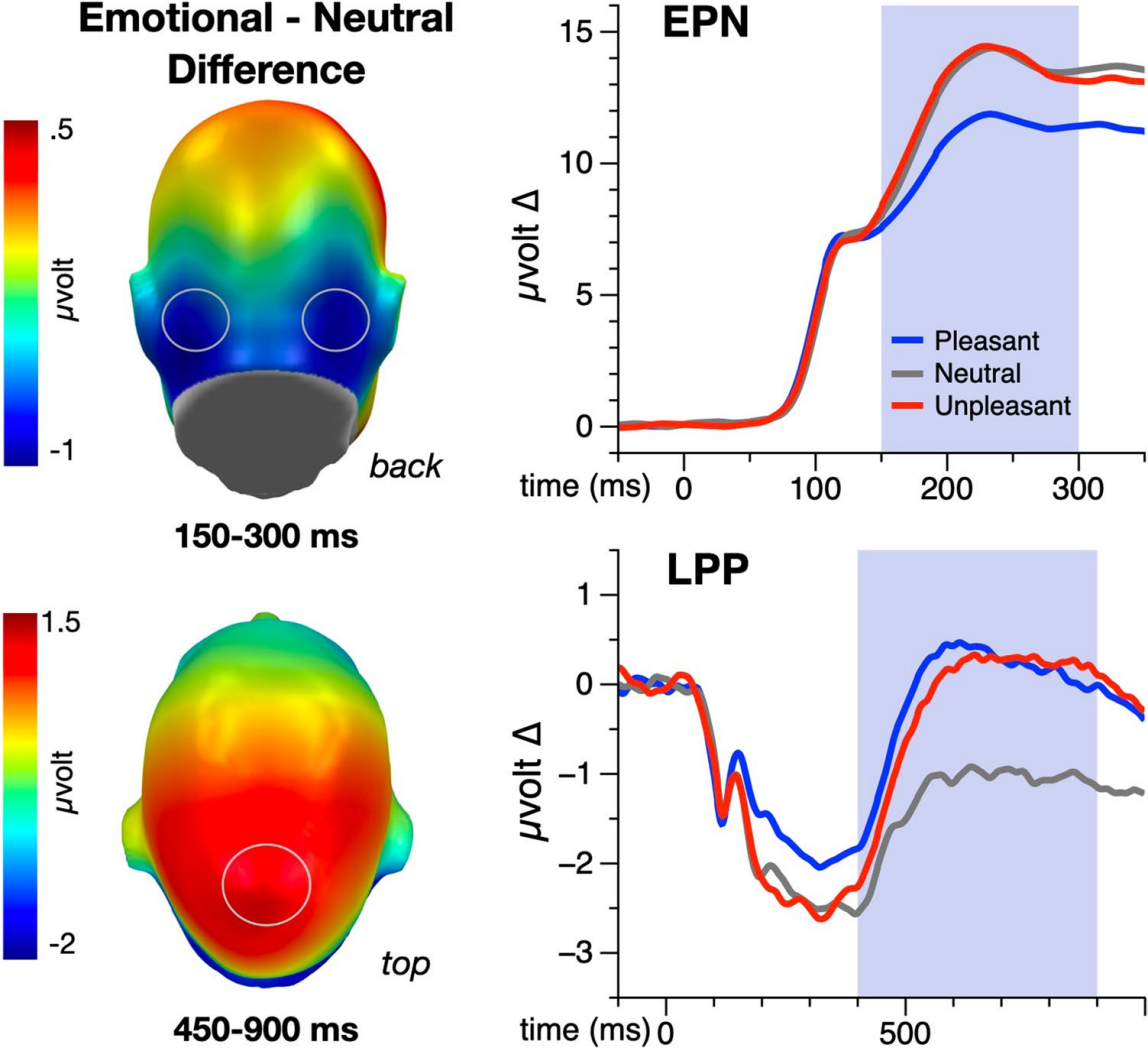
ERP difference waves (emotional–neutral) for the early posterior negativity (EPN, 150–300 ms) and late positive potential (LPP, 450–900 ms). Waveforms and topographies are averaged across the three image categories (objects, people, and landscapes) and shown separately for each emotional valence condition

**Fig. 3:**
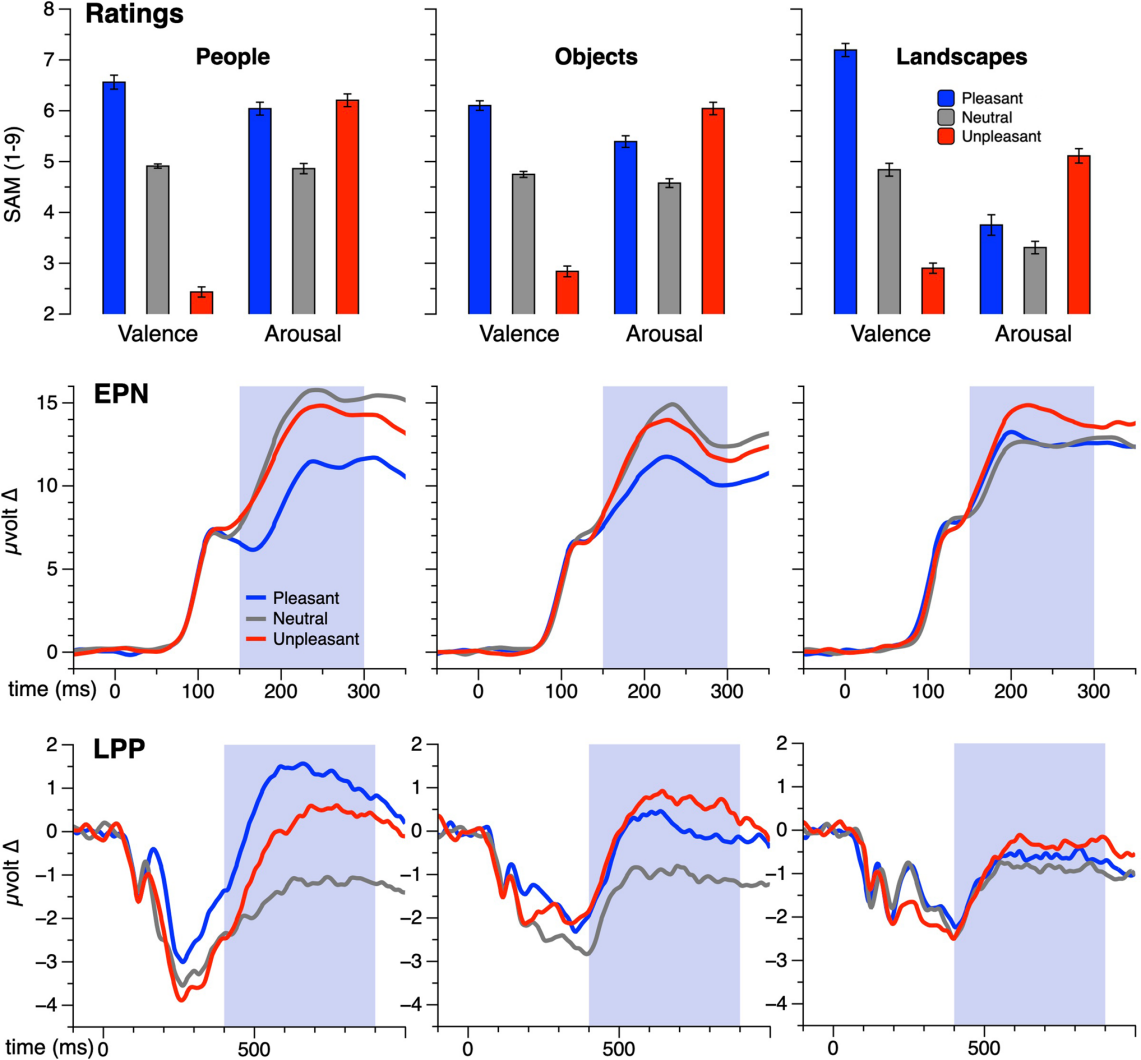
Subjective ratings and ERP responses by category. Top row: Mean valence and arousal ratings for People, Objects, and Landscapes (9-point SAM scales). Bottom row: ERP waveforms for the EPN (150–300 ms) and LPP (450–900 ms) for Pleasant, Neutral, and Unpleasant stimuli within each category. Shaded regions mark analysis windows

Shown in Fig. 2, EPN amplitude was strongly modulated by scene valence *F*(2, 152) = 185.44, *p* < .001,* η*^*2*^ = .709, and more modestly affected by scene content *F*(2, 152) = 8.02, *p* < .001, *η*^*2*^ = .096. Shown in Fig. 3, there was also a large interaction of emotion and content, *F*(4, 304) = 69.026,* p* < .001, *η*^*2*^ = .476. Bonferroni-corrected post-hoc comparisons are listed in Tables 2 and 3, which show that emotional people and object scenes enhance the EPN similarly, with the greatest modulation during pleasant, relative to neutral scenes, (*ps* < .001), and reliable enhancement of EPN when viewing unpleasant versus neutral people scenes (*p* < .001). Although pleasant people and object scenes evoked larger EPN modulation than unpleasant people and object scenes (Fig. 3; Tables 2 and 3), their difference topographies (pleasant vs. neutral and unpleasant vs. neutral) were consistent. The pattern of modulation during landscape scenes was much different, and inconsistent with prior findings in the literature. Specifically, there was greater voltage negativity during neutral relative to unpleasant landscapes (*p* < .001), and no differentiation of the EPN during pleasant and neutral landscape scenes (*p* > .05). In brief, emotional people and object scenes show modulation consistent with past work, while landscapes scenes do not (Table 4).

**Table 2:** Comparisons of ERP amplitudes across emotion within category

EPN					
Content	Emotion (I)	Emotion (J)	Difference (I-J)	Standard error	*p*
Landscapes	Neutral	Pleasant	− 0.333	0.188	ns
	Neutral	Unpleasant	− 1.633	0.2	< .001
	Pleasant	Unpleasant	−1.3	0.177	< .001
Objects	Neutral	Pleasant	2.317	0.211	< .001
	Neutral	Unpleasant	0.404	0.206	ns
	Pleasant	Unpleasant	− 1.912	0.223	< .001
People	Neutral	Pleasant	4.048	0.218	< .001
	Neutral	Unpleasant	0.729	0.174	< .001
	Pleasant	Unpleasant	− 3.32	0.203	< .001
LPP					
Content	Emotion (I)	Emotion (J)	Difference (I-J)	Standard error	*p*
Landscapes	Neutral	Pleasant	− 0.229	0.139	ns
	Neutral	Unpleasant	− 0.418	0.181	ns
	Pleasant	Unpleasant	− 0.189	0.146	ns
Objects	Neutral	Pleasant	− 1.042	0.14	< .001
	Neutral	Unpleasant	− 1.509	0.142	< .001
	Pleasant	Unpleasant	− 0.467	0.138	< .01
People	Neutral	Pleasant	− 2.295	0.183	< .001
	Neutral	Unpleasant	− 1.252	0.18	< .001
	Pleasant	Unpleasant	1.043	0.181	< .001

**Table 3:** Comparisons of ERP amplitudes across category within emotion

EPN					
Emotion	Content (I)	Content (J)	Difference (I-J)	Standard error	*p*
Neutral	Landscapes	Objects	− 0.908	0.251	< .01
	Landscapes	People	− 1.781	0.268	< .001
	Objects	People	− 0.873	0.189	< .001
Pleasant	Landscapes	Objects	1.742	0.219	< .001
	Landscapes	People	2.6	0.269	< .001
	Objects	People	0.858	0.237	< .01
Unpleasant	Landscapes	Objects	1.129	0.199	< .001
	Landscapes	People	0.58	0.193	< .05
	Objects	People	− 0.549	0.225	ns
LPP					
Emotion	Content (I)	Content (J)	Difference (I-J)	Standard error	*p*
Neutral	Landscapes	Objects	0.137	0.187	ns
	Landscapes	People	0.363	0.208	ns
	Objects	People	0.226	0.151	ns
Pleasant	Landscapes	Objects	− 0.675	0.159	< .001
	Landscapes	People	− 1.702	0.184	< .001
	Objects	People	− 1.027	0.168	< .001
Unpleasant	Landscapes	Objects	− 0.953	0.157	< .001
	Landscapes	People	− 0.471	0.185	< .05
	Objects	People	0.483	0.162	< .05

**Table 4:** Paired *t*-tests for pleasant/unpleasant vs. neutral content categories

Measure	Content	Pleasant (M ± SD)	Neutral (M ± SD)	Unpleasant (M ± SD)	Pleasant vs. neutral	Unpleasant vs. neutral
LPP	Landscapes	− 0.84 ± 1.46	− 1.07 ± 1.34	− 0.65 ± 1.69	*t*(76) = 1.7, ns	*t*(76) = 2.3, ns
	People	0.86 ± 1.87	− 1.44 ± 1.91	− 0.18 ± 2.27	*t*(76) = 12.5, *p* < .001	*t*(76) = 7.0, *p* < .001
	Objects	− 0.17 ± 1.76	− 1.21 ± 1.82	0.30 ± 1.92	*t*(76) = 7.5, *p* < .001	*t*(76) = 10.6,* p* < .001
EPN	Landscapes	12.13 ± 4.91	11.8 ± 4.94	13.43 ± 4.97	*t*(76) = 1.8, ns	*t*(76) = 8.1, *p* < .001
	People	9.53 ± 4.95	13.58 ± 5.20	12.85 ± 5.21	*t*(76) = − 18.6, *p* < .001	*t*(76) = − 4.2,* p* < .001
	Objects	10.39 ± 5.02	12.71 ± 5.18	12.30 ± 5.02	*t*(76) = − 11, *p* < .001	*t*(76) = − 1.96, ns

### Onset ERP modulation—late positive potential

Shown in Fig. 2, LPP amplitude was strongly modulated by scene valence, *F*(2, 152) = 98.006,* p* < .001, *η*^*2*^ = .563, and more modestly affected by scene content, *F*(1.71, 130.003) = 15.413, *p* < .001, *η*^*2*^ = .169. Shown in Fig. 3, there was also a large interaction of emotion and content, *F*(4, 304) = 26.865, *p* < .001, *η*^*2*^ = .261. Post-hoc tests showed that LPP amplitudes during neutral scenes were very similar across landscapes, objects, and people (*ps* > .05). Thus, the interaction was driven by enhanced positivity by emotional scenes. Emotional people and object scenes show similar LPP modulation, with greater positivity during pleasant and unpleasant, relative to neutral scenes (*ps* < .001), although the relative impact of pleasant and unpleasant scenes differed. Emotional landscapes did not significantly enhance LPP positivity relative to neutral landscapes (*ps* > .05), despite reliably enhanced arousal ratings. Descriptive statistics for Bonferroni-corrected post-hoc comparisons (means, SEs, and corrected *p* values) are reported in Tables 2–3. In sum, like the EPN, emotional people and object scenes show LPP modulation consistent with past work, while landscape scenes do not.

### EPN modulation—arousal-balanced scene subset

**Fig. 4:**
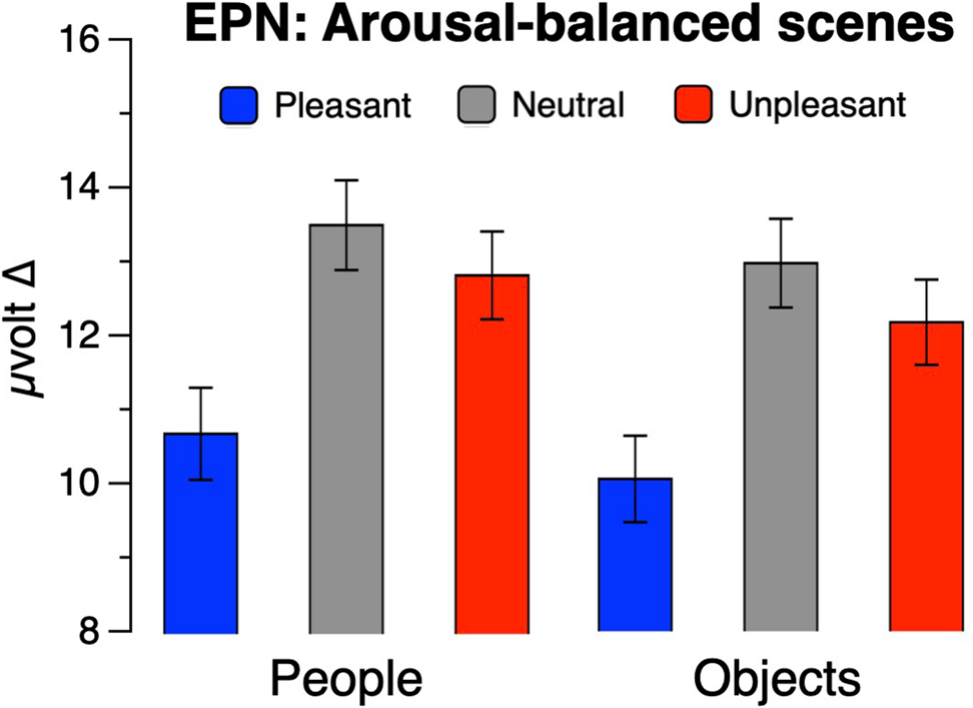
Mean EPN amplitudes (150–300 ms) for arousal balanced scenes across categories (People, Objects, Landscapes). Bars represent Pleasant (blue), Neutral (gray), and Unpleasant (red) conditions. Error bars indicate SEM

The scene stimuli used in the study were chosen to increase the likelihood of receiving equivalent ratings of arousal from our sample, which required the use of moderately arousing people scenes, and the most arousing object and landscape scenes available. While this effort was mostly successful for people and object scenes, landscape scenes were rated as considerably less arousing than other contents. To separate the contribution of animacy (people vs. objects) and rated arousal on emotional modulation of the EPN, we used the ratings data from our EEG sample to select a subset of people and object scenes that yielded equivalent arousal ratings. This subset was selected by iteratively removing highly arousing people scenes and weakly arousing object scenes until the mean arousal ratings by content did not reliably differ (*p* > .20), which resulted in 44 of 60 people scenes and 44 of 60 object scenes in the subset. Shown in Fig. 4, emotional modulation of the EPN did not interact with content (people vs. objects) in response to this arousal-matched scene subset. Therefore, when equivalently arousing scenes are viewed, the EPN is modulated consistently across scenes featuring people and scenes featuring objects, and animacy is not a factor.

## Discussion

The great majority of emotional perception studies measuring the EPN and LPP have shown these ERPs to be similarly modulated by emotionally arousing content, both pleasant and unpleasant (Olofsson et al., 2008; Schupp et al., 2006; Weinberg & Hajcak, 2010). Considering the obvious differences in latency, polarity, and scalp topography, these ERPs are of course likely to represent distinct aspects of perceptual and emotional processing. The primary purpose of this study was to assess the possibility that the EPN may be specifically sensitive to bodies, which was addressed by comparing EPN modulation to matched sets of emotional people and objects. The results suggest that this is not the case, as a consistent pattern of EPN modulation was evident across 60 people scenes and 60 object scenes. Moreover, after selecting a subset of 44 people and 44 object scenes that received uniform arousal ratings from our sample of 77 participants, EPN amplitudes during pleasant, neutral, and unpleasant scenes were equivalent across people and objects. Because this experiment was powered to detect small effect sizes, we are reasonably confident that this absence of effect is reliable.

Consistent with the latency of the EPN, during which basic scene elements are deciphered (Baker & Kravitz, 2024), the EPN has been shown to be enhanced during simple figure/ground, relative to more complex scenes, while the LPP is not (Bradley et al., 2007; Löw et al., 2013; Wiens, 2011). The EPN is also larger during scenes depicting people (Farkas et al., 2020; Löw et al., 2013; Thierry et al., 2006), while the LPP is consistently associated with arousing scenes, independent of depicted content (Bradley et al., 2007; Ferrari et al., 2016; Weinberg et al., 2021). Source localization analyses of the EPN using dense-array EEG suggests a lateral occipital focus (Junghöfer et al., 2001; Schupp et al., 2006), although the amplitude of the brief EPN has not been shown to correlate with increases in fMRI BOLD signal from lateral occipital, superior parietal, or inferior temporal visual structures to individual scene stimuli (Sabatinelli et al., 2013). In contrast, the extended slow-wave LPP appears to reflect a composite of processing across multiple visual structures and anterior regions of the brain (Keil et al., 2009; Liu et al., 2012; Moratti et al., 2011).

Regarding emotional scene content, while highly arousing scenes enhance both the EPN and the LPP, the correlation amplitudes across a given set of experimental stimuli is modest, and primarily attributable to consistently weak modulation by neutral scenes (Sabatinelli et al., 2013). In a study in which erotica and mutilation scenes were balanced in rated arousal and perceptual complexity, the LPP tracked rated arousal, while the EPN was more strongly driven by pleasant content (Frank & Sabatinelli, 2019). This suggested the possibility of a pleasure bias in the EPN, an effect that had been reported before (De Cesarei & Codispoti, 2006; Flaisch et al., 2008; Weinberg & Hajcak, 2010), but not discussed or attributed to idiosyncratic differences in arousal of the experimental scenes.

A study assessed the possibility of an EPN pleasure bias, in which EPN amplitudes in response to erotic scenes were compared to EPN amplitudes during equivalently arousing and even more pleasant scenes of sports victories (Farkas et al., 2020). In contrast with a pleasure bias, victory scenes weakly modulated the EPN relative to erotica. This effect suggested that the EPN may reflect a specific bias toward sexual cues and led to a control study to test the possibility that exposed bodies, and not sexual cues might enhance the EPN, perhaps reflecting activity in lateral occipital cortex associated with body processing (Downing et al., 2001; Urgesi et al., 2007). The control condition recorded the EPN during erotic scenes as well as scenes of nudist couples in nonsexual contexts and led to the surprising result of enhanced EPN amplitudes during nudist scene perception, despite much weaker arousal and valence ratings (Farkas et al., 2020). Finally, an expanded study was conducted to replicate and extend this finding, in which the EPN was found to be highly sensitive to upright, exposed bodies in scenes, independent of rated arousal, distinct from modulation of the LPP (Farkas & Sabatinelli, 2023).

The apparent body-part sensitivity of the EPN identified in these prior studies led to the current experiment assessing whether emotional modulation of the EPN is enhanced by (or possibly dependent on) people depicted in scene stimuli. Instead, as described above, people and object scenes that were rated as equivalently arousing modulated the EPN to the same extent (Fig. 4). Thus, when arousal is held constant, animacy does not appear to be an independent modulator of the EPN.

These results should not be interpreted as evidence that stimulus animacy should be discounted as a modulator of the EPN in all cases. Animacy detection is likely a feature of the primate visual system that evolved to enable the fine categorization of subtle cues for the purposes of predator avoidance (Epstein & Baker, 2019; Kravitz et al., 2013; Rudebeck et al., 2017), and several fMRI studies have identified a medial/lateral separation of object/animal-driven activity in ventral visual cortex (Graham et al., 2010; Sha et al., 2015; Watson & Andrews, 2024). Yet in this attempt to characterize the impact of scene animacy, a key design element was the use of moderately arousing people scenes, chosen in the knowledge that animate scenes typically evoke higher arousal ratings than object scenes. Thus, the parallel effect sizes seen in the current results may be limited to these or similar scene stimuli. Future work comparing the impact of emotional object scenes against more evocative animate contents, such as erotica and intense threat scenes may reveal how these motivationally relevant contents differentially influence ERP modulation.

Although not a focus of the current study, a consistent offset in EPN modulation was apparent favoring pleasant, relative to unpleasant content in both scenes featuring people and scenes featuring objects. This pattern is consistent with earlier work discussed above and remains unresolved. The association of pleasantness and EPN modulation is not straightforward or consistent. For example, when participants view scenes depicting sports victory, which they rate as extremely pleasant and highly arousing, the evoked EPN is considerably weaker than that evoked by erotica, which they rate as equally arousing and *less* pleasant (Farkas et al., 2020; Farkas & Sabatinelli, 2023). Future studies that target this potential pleasure bias will require careful design and analyses to investigate multiple interacting scene features and their effects on the EPN, LPP, and other physiological and experiential measures.

The scene categories compared in this study were not balanced for color or spatial frequency. While the scene set was assembled to be comparable in composition (Bradley et al., 2007; Wiens et al., 2011) and statistically equivalent in luminance and 90% quality JPEG file size, not all scene features were explicitly controlled. The first study to identify the EPN (Junghöfer et al., 2001) included color and spatial frequency as predictors and identified no relationship. High and low spatial frequency was also not associated with EPN amplitude in a recent study using a wide array of emotional and neutral scene content (Farkas & Sabatinelli, 2023). Future studies will be needed to explicitly control the potential impact of these features across animate and inanimate content.

One practical outcome from this research relates to the use of landscape scenes in EEG studies of emotional perception, typically as neutral 'control' stimuli. This practice does not appear to be appropriate, because no evidence of LPP modulation was identified between emotional and neutral landscape scenes, despite arousal ratings that might suggest such modulation might be present. This lack of modulation is not secondary to any levels difference, as neutral scenes of all contents (people, objects, landscapes) evoked the same level of LPP positivity. Future studies should avoid the use of landscapes as comparators with people and object scenes, or at least balance the number of landscapes across emotional and neutral conditions.

In summary, consistent with past work, the EPN appears to be consistently modulated by the emotional intensity of scene stimuli, whether the scene depicts an emotionally arousing person or an emotionally arousing object. This extends the utility of the EPN as a measure of the processing of emotional cues in general, and differentiates it from overlapping face and body sensitive ERP components that occur in this latency window and in close proximity on the scalp. The EPN therefore appears to represent an early recognition of emotional content, perhaps with a voltage field that is intermixed with concurrent regional sensitivities to specific visual features that may or may not reflect emotional quality, such as perceptual complexity and the presence of bodies in scenes. Future studies that include a more variable range of scene contents beyond those associated with basic motivational drives, such as sex and violence, may enable a more refined definition of how the EPN (and LPP) index naturalistic scene discrimination and emotional engagement.

## Data Availability

The datasets and materials generated and analyzed during the current study are available from the corresponding author upon reasonable request.

## Code availability

The code used in this study is available from the corresponding author upon reasonable request.

## Declarations

### Open practices statement

The data and materials for the experiment are available from the corresponding author upon reasonable request. The experiment was not preregistered.
